# Genome-Wide Detection of Runs of Homozygosity in Laiwu Pigs Revealed by Sequencing Data

**DOI:** 10.3389/fgene.2021.629966

**Published:** 2021-04-29

**Authors:** Yifei Fang, Xinyu Hao, Zhong Xu, Hao Sun, Qingbo Zhao, Rui Cao, Zhe Zhang, Peipei Ma, Yanxiao Sun, Zengmin Qi, Qingkui Wei, Qishan Wang, Yuchun Pan

**Affiliations:** ^1^Department of Animal Science, School of Agriculture and Biology, Shanghai Jiao Tong University, Shanghai, China; ^2^Department of Animal Science, College of Animal Sciences, Zhejiang University, Hangzhou, China; ^3^Breeding Company of Pigs, Laiwu, China

**Keywords:** runs of homozygosity, inbreeding coefficient, candidate genes, selection, Laiwu pig

## Abstract

Laiwu pigs, distinguished by their high intramuscular fat of 7–9%, is an indigenous pig breed of China, and recent studies also found that Laiwu pigs showed high resistance to Porcine circovirus type 2. However, with the introduction of commercial varieties, the population of Laiwu pigs has declined, and some lineages have disappeared, which could result in inbreeding. Runs of homozygosity (ROH) can be used as a good measure of individual inbreeding status and is also normally used to detect selection signatures so as to map the candidate genes associated with economically important traits. In this study, we used data from Genotyping by Genome Reducing and Sequencing to investigate the number, length, coverage, and distribution patterns of ROH in 93 Chinese Laiwu pigs and identified genomic regions with a high ROH frequency. The average inbreeding coefficient calculated by pedigree was 0.021, whereas that estimated by all detected ROH segments was 0.133. Covering 13.4% of the whole genome, a total of 7,508 ROH segments longer than 1 Mb were detected, whose average length was 3.76 Mb, and short segments (1–5 Mb) dominated. For individuals, the coverage was in the range between 0.56 and 36.86%. For chromosomes, SSC6 had the largest number (*n* = 688), and the number of ROH in SSC12 was the lowest (*n* = 215). Thirteen ROH islands were detected in our study, and 86 genes were found within those regions. Some of these genes were correlated with economically important traits, such as meat quality (*ECI1*, *LRP12*, *NDUFA4L2*, *GIL1*, and *LYZ*), immunity capacity (*IL23A, STAT2*, *STAT6*, *TBK1*, *IFNG*, and *ITH2*), production (*DCSTAMP*, *RDH16*, and *GDF11*), and reproduction (*ODF1* and *CDK2*). A total of six significant Gene Ontology terms and nine significant Kyoto Encyclopedia of Genes and Genomes (KEGG) pathways were identified, most of which were correlated with disease resistance and biosynthesis processes, and one KEGG pathway was related to lipid metabolism. In addition, we aligned all of the ROH islands to the pig quantitative trait loci (QTL) database and finally found eight QTL related to the intramuscular fat trait. These results may help us understand the characteristics of Laiwu pigs and provide insight for future breeding strategies.

## Introduction

Laiwu pigs, a precious Chinese indigenous pig breed, are mainly distributed in Shandong Province, China. They are distinguished by a good meat quality, especially a high intramuscular fat (IMF) content of 9–12%, compared with the major commercial lean pig breeds (Yang et al., 2015). Recent studies have also found that Laiwu pigs showed high resistance to certain infectious diseases, such as Porcine circovirus type 2 (PCV2) (Li et al., 2016). These breed-specific features of Laiwu pigs are the consequences of natural and human-mediated selection. Therefore, detection of selection signatures across Laiwu pigs’ genome may help us to reveal the genetic mechanism underlying the process of IMF deposition, as well as the genes involved in resistance to PCV2.

In addition, during the last 40 years, with the introduction of western pig breeds such as Duroc and Large White pigs, the Laiwu pig population has shrunk, and some lineages have disappeared. This caused a decrease in both the nucleotide diversity and additive genetic variance in the Laiwu pig populations and led to inbreeding, which can undermine fitness traits due to the expression of highly homozygous detrimental recessive alleles (Howard et al., 2017). Thus, monitoring the genetic inbreeding levels within populations is of great importance in the genetic protection and improvement of Laiwu pigs.

The classical method for estimating the inbreeding level of a population is by calculating the inbreeding coefficient through pedigree-based information (Wright, 1922), but pedigree errors are not rare in livestock populations (Ron et al., 1996). Therefore, several more efficient alternative methods based on genome-wide sequencing data have been applied to calculate inbreeding coefficients, including ROH-based inbreeding coefficients (*F*_ROH_) (McQuillan et al., 2008), which has recently become a popular method and has been successfully used in studying genomic inbreeding for various kinds of livestock populations (Ferencakovic et al., 2011; Szmatoła et al., 2019; Zhan et al., 2020). Runs of homozygosity (ROH) are defined as continuous homozygous DNA segments in diploid genomes, which are a result of the inheritance of identical haplotypes under the condition that both parents descended from a common ancestor (Gibson et al., 2006). Inbreeding is one of the main reasons for the formation of ROH (Curik et al., 2014), indicating that ROH can be used as a good measure of individual inbreeding status. Meanwhile, it has been found that both ROH number and length can provide insights into genome-wide inbreeding levels (Purfield et al., 2012), and the length of the identified ROH segments could also reflect the number of generations since inbreeding occurred (McQuillan et al., 2008).

In addition to inbreeding, other population events can also result in the formation of ROH, including genetic drift, population bottleneck, and selection (Frankham, 1996). Thus, the population history, structure, and germplasm features could be revealed by the identification and characterization of ROH (MacLeod et al., 2013; Peripolli et al., 2017). It has been found that the distribution pattern of ROH is not random across a genome (Bosse et al., 2012), and genomic regions with the highest frequency of ROH are named “ROH islands” or “ROH hotspots” (Nothnagel et al., 2010; Pemberton et al., 2012a). ROH islands are observed to be shared among individuals, which is probably a ramification of selection, as genomic regions harboring selection signatures usually overlap with ROH islands (Zhang et al., 2015). As positive directional selection can further reduce genetic diversity and increase homozygosity, thus contributing to the formation of ROH islands, genomic regions under selection are more likely to overlap with ROH islands than the rest of the genome (Pemberton et al., 2012a). Therefore, the analysis of ROH islands is commonly used to reveal putative genomic regions under selection and to disclose the genetic background of economically important traits among livestock populations (Zhang et al., 2015; Xiao et al., 2017; Onzima et al., 2018). In recent years, ROH detection has been used commonly in pigs, but few studies on ROH analysis of Laiwu pigs can be found.

The purposes of this study were to detect and characterize the ROH patterns in Laiwu pig populations using the data obtained by the Genotyping by Genome Reducing and Sequencing (GGRS) (Chen et al., 2013) method, as well as to investigate ROH islands that could contain candidate genes related to the characterized traits. Furthermore, we aimed to compare inbreeding coefficients estimated using ROH, pedigree, and single-nucleotide polymorphism (SNP) information.

## Materials and Methods

### DNA Sampling and Sequencing

According to the genealogical records, 93 unrelated or distantly related Laiwu pigs (including 17 male pigs and 76 female pigs) were selected from the National Conservation Farm for Laiwu Pigs in Shandong Province, China. DNA samples were extracted from their ear tissues with commercial kits (Lifefeng Biotech, Co., Ltd., Shanghai, China) and then genotyped using the GGRS method (Chen et al., 2013).

The GGRS method includes the following steps: First, the DNA sequencing libraries were prepared. Next, they were sequenced using paired-end (2 × 150 bp) sequencing methodology with an Illumina HiSeq2000 sequencer. Quality control for this procedure was conducted by filtering raw reads, as reads with base average quality scores less than 30 (an error rate of base calling of 0.1%) were excluded using NGS QC Toolkit v2.3 (Patel and Jain, 2012). The other remaining reads were then aligned to the pig genome reference (Sscrofa11.1) by BWA software (Li and Durbin, 2010). Aligned reads were used for further SNP identification and genotyping, SAMTOOLS v1.4 was used with default settings in this procedure, and missing SNP genotypes were imputed with BEAGLE v4.1 (Browning and Browning, 2016). After imputation, quality control was adopted again by applying the following criteria: all SNPs were detected in more than 30% of the samples and had no less than six-fold sequencing depth on average, as well as a minor allele frequency (MAF) ≥5%. In addition, reads mapped to the mitochondrial and sex chromosomes were excluded due to the high rates of discordance during the process of inheritance.

### Estimation of Genetic Diversity

We calculated the observed heterozygosity (*H*_o_) and expected heterozygosity (*H*_e_) by PLINK v1.9 (Purcell et al., 2007). According to the classical theory of quantitative genetics proposed by Falconer D.S., if a population always keeps the same amounts of offspring for each family during the process of breed conservation, the *N*_e_ can be estimated using the following formula (Falconer, 1962):

(1) $$N_{e}=\frac{16\,N_{m}\,N_{f}}{N_{f}+N_{m}}$$

in which Ne is the effective population size, and *N*_m_ and *N*_f_ are the number of male and female animals in this population, respectively.

### Runs of Homozygosity Detection

ROH for each animal were estimated using *“*- *homozyg”* of PLINK v1.9 (Purcell et al., 2007) with the default parameter: –homozyg-snp 100, –homozyg-window-snp 50, –homozyg-window-missing 5, and so on. We used a sliding window consisting of an appointed number of SNPs to scan through each sample’s whole genome to locate the homozygous regions. One Mb was set as an ROH length threshold to exclude short homozygous segments induced by LD effects. Other criteria applied for the identification of ROH were as follows (Peripolli et al., 2018): (1) 50 SNPs were contained in each sliding window; (2) an ROH consisted of no less than 100 consecutive SNPs; (3) the density should be higher than one SNP per 50 kb; (4) SNPs with missing genotypes less than five and with a heterozygous genotype less than one were allowed in an ROH due to genotyping error. All homozygous segments filtered were classified into three length classes: 1–5, 5–10, and >10 Mb, identified as ROH_1–5 Mb_, ROH_5–10 Mb_, and ROH_>10 Mb_, respectively.

### Inbreeding Coefficients

We used several different methods to calculate inbreeding coefficients according to the pedigree and genomic information, and these inbreeding coefficients were further compared by Pearson’s correlation analysis. The R package “pedigree” was used to estimate the inbreeding coefficients based on pedigree (*F*_PED_) for all individuals (Coster and Coster, 2010). Inbreeding coefficients were calculated according to the genomic information, which includes those estimated from ROH (*F*_ROH_) (McQuillan et al., 2008) and from SNP information (*F*_SNP_). *F*_ROH_ was calculated according to the following formula:

(2) $$F_{\text{ROH}}=\frac{L_{\text{ROH}}}{L_{\text{auto}}}$$

where *L*_ROH_ is defined as the total length of the genome covered by all ROH segments for each individual, and *L*_auto_ is the length of the sequenced genome, which equaled 2.26 Gb in this research. Following the study of Xu et al. (2019), we divided *F*_ROH_ into four types of estimates based on the length of the observed ROH: *F*_ROH_all_ (>1 Mb), *F*_ROH1–5 Mb_ (1–5 Mb), *F*_ROH5–10 Mb_ (5–10 Mb), and *F*_ROH>10 Mb_ (>10 Mb). It has been reported that a lower limit of ROH length of 1 Mb has a genetic distance of 1 cM approximately (Zanella et al., 2016), which allows us to use the *F*_ROH _all_ (>1 Mb) to trace the ancestral inbreeding events that occurred 50 generations ago. Similarly, the corresponding spans for the last three F_ROH_ estimates are 10 to 50 generations, 5 to 10 generations, and 5 generations.

GCTA software was used to obtain the three estimates of inbreeding coefficients based on SNP (*F*_SNP1_, *F*_SNP2_, and *F*_SNP3_) with the option “*-ibc*” (Yang et al., 2011): they were derived based on the variance of the additive genotypes (VanRaden, 2008), the degree of homozygous excess (Wright, 1948), and the correlation between the uniting gametes (Wright, 1922). Discussed hereafter are the formulae used:

(3) $$F_{\text{SNP1}}=\frac{1}{n}\sum_{i=1}^{n}\frac{{\left(Y_{i}-2p_{i}\right)}^{2}}{h_{i}}-1$$

(4) $$F_{\text{SNP2}}=1-\frac{1}{n}\sum_{i=1}^{n}\frac{Y_{i}(2-Y_{i})}{h_{i}}$$

(5) $$F_{\text{SNP3}}=\frac{1}{n}\sum_{i=1}^{n}\frac{Y_{I}^{2}-Y_{i}(1+2p_{i})+2p_{i}^{2}}{h_{i}}$$

In which Y_i_ is the amount of the reference allele copies at the i-th SNP locus, p_i_ refers to the frequency of the allele at the i-th SNP locus for this individual, *h*_i_ is calculated as: *h*_i_ = 2p_i_(1−*p*_i_) and n is the amount of SNPs.

### Detection of Runs of Homozygosity Islands and Candidate Genes

To detect the ROH islands, we calculated the frequency of occurrences within the ROH regions of each SNP across the individuals and made a Manhattan figure by plotting these values in conformity with the position of each SNP on chromosomes. The SNPs in the top 1% of the frequency of occurrence were selected as a hint of a potential ROH island (Pemberton et al., 2012b). A chain of adjoining selected SNPs can merge to integrate an ROH island. Genes that were fully or partially contained by these ROH islands were identified and annotated using the University of California, Santa Cruz Genome Browser^1^. Further analysis of gene functions was performed using the Kyoto Encyclopedia of Genes and Genomes (KEGG) pathway and Gene Ontology (GO) enrichment analyses by DAVID 6.8^2^, and terms with a *p*-value greater than 0.05 were filtered.

## Results

### DNA Sequencing and Genetic Diversity

**FIGURE 1:**
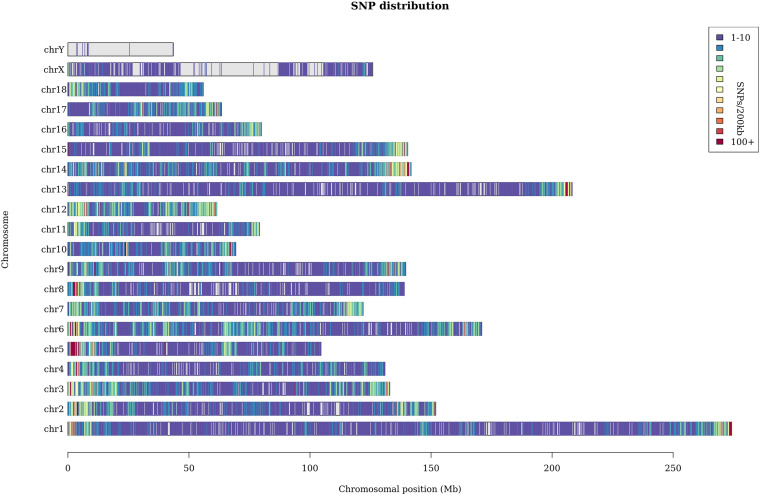
Distribution of the sequenced SNPs on all chromosomes. The *y*-axis represents chromosomes, and the *x*-axis represents the corresponding chromosomal position (Mb). Different colors of each 200-kb genome block denote the number of SNPs.

A total of 369 million clean reads were obtained, and each individual pig had approximately 4.75 million clean reads. The average sequencing coverage of the entire genome was 2.5%, and the average sequencing depth was 8×. With the invalid data filtered out, we finally obtained 182,021 informative SNPs. The sequenced SNPs were distributed evenly along the genome (Figure 1), and the average density was approximately 1 SNP per 10.3 Kb. We mapped these SNPs to the Ensemble pig gene annotation set (Ensemble release 92) and found that 91,064 SNPs were within gene regions, including 3,218 SNPs within exons and 9,817 within untranslated regions. The result shows that approximately 50% SNPs were within gene regions, but only 1.7% of SNPs were within exons. This result is unexpected. GGRS libraries were not designed to target polymorphisms within genes, so this proportion seems relatively high. Similar results have been reported in Jinhua pigs (Xu et al., 2019). The average *H*_o_ and *H*_e_ of this Laiwu pig population were 0.27 and 0.38, respectively. The value of *H*_o_ is lower than the result reported before (0.284), whereas *H*_e_ is higher (0.281) (Wang et al., 2021). In addition, the *N*_e_ of this population was 52.6, which suggests it is quite likely that inbreeding has happened.

### Inbreeding Coefficients Estimate

The inbreeding coefficient of each individual estimation based on pedigree, ROH of different lengths, and the SNPs are shown in Table 1. The average inbreeding coefficient estimates obtained from different lengths of ROH segments follow this order, *F*_ROH>10 Mb_ < *F*_ROH5–10 Mb_ < *F*_ROH1–5 Mb._ Compared with them, *F*_PED_, the average inbreeding coefficient calculated from the pedigree, was the lowest (0.021).

**TABLE 1:** Descriptive statistics for the eight types of inbreeding coefficient estimates calculated from the pedigree (*F*_PED_), the identified ROH (*F*_ROH_ all_, *F*_ROH1–5 Mb_, *F*_ROH5–10 Mb_, and *F*_ROH > 10 Mb_), and the SNP information (*F*_SNP1_, *F*_SNP2_, and *F*_SNP3_).

Inbreeding coefficient	Mean	Min	Max	SD
*F*_PED_	0.021	0.000	0.250	0.040
*F*_ROH1–5 Mb_	0.065	0.003	0.123	0.027
*F*_ROH5–10 Mb_	0.039	0.002	0.109	0.023
*F*_ROH>10 Mb_	0.038	0.005	0.157	0.028
*F*_ROH–all_	0.133	0.006	0.369	0.071
*F*_SNP1_	0.276	0.052	0.792	0.129
*F*_SNP2_	0.276	0.230	0.513	0.276
*F*_SNP3_	0.154	0.141	0.554	0.123

*Min, minimum; Max, maximum; SD, standard deviation.*

The correlation coefficients among all of the results estimated by different methods are shown in Table 2. The correlation between inbreeding coefficients estimated by the pedigree and the genomic data was weak (varied from −0.188 to 0.055). The correlation among all of the estimates calculated based on the ROH information was strong. *F*_ROH__ was strongly correlated with the other ROH-based estimates, and the correlation coefficients between *F*_ROH_ all_ and *F*_ROH1–5 Mb_ were the highest (0.910), whereas the weakest correlation among the ROH-based estimates was between *F*_5–10 Mb_ and *F*_>10 Mb_, which was 0.593. The correlation among all the values calculated by the SNPs was strong and statistically significant. However, the correlations between the estimates based on the SNPs and ROH data were relatively strong, except for *F*_SNP2_, which was significantly correlated with all of the ROH-based inbreeding coefficients.

**TABLE 2:** Correlation coefficients (lower panel) among eight types of inbreeding coefficient estimates (*F*_PED_, *F*_ROH1–5 Mb_, *F*_ROH5–10 Mb_, *F*_ROH_ **_>_**_10 Mb_, *F*_ROH_ all_, *F*_SNP1_, *F*_SNP2_, and *F*_SNP3_).

Correlation	*F*_PED_	*F*_ROH1–5 Mb_	*F*_ROH5–10 Mb_	*F*_ROH > 10 Mb_	*F*_ROH_all_	*F*_SNP1_	*F*_SNP2_	*F*_SNP3_
*F*_PED_	1							
*F*_ROH1–5 Mb_	0.029	1						
*F*_ROH5–10 Mb_	–0.095	0.701**	1					
*F*_ROH>10 Mb_	0.035	0.645**	0.593**	1				
*F*_ROH_all_	0.055	0.910**	0.810**	0.870**	1			
*F*_SNP1_	–0.051	0.106	0.170	0.108	0.129	1		
*F*_SNP2_	–0.188	0.447**	0.364**	0.325**	0.422**	0.508**	1	
*F*_SNP3_	–0.144	0.335**	0.317	0.260	0.332**	0.842**	0.892**	1

**Significantly different *p* < 0.01.

### Genomic Distribution of Runs of Homozygosity

**FIGURE 2:**
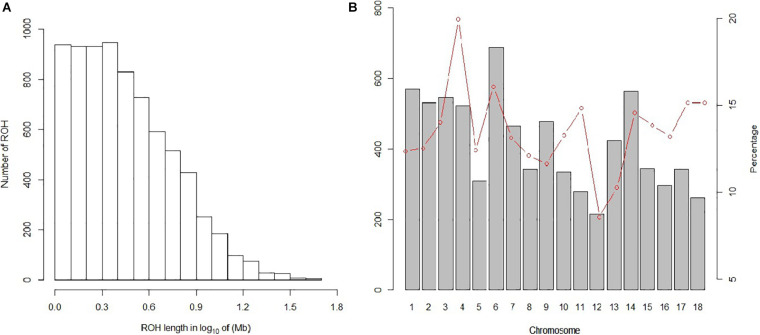
Distribution of the ROH. **(A)** Length distribution of the ROH. *X*-axis represents the length of the ROH (Mb) using a base-10 log scale. *Y*-axis represents the number of ROH for different ROH lengths. **(B)** Bars and the red dotted line represent the number of ROH and the ROH coverage, respectively, on each chromosome.

In our study, 7,508 ROH segments were found in 93 individuals, with an average length of 3.76 Mb. The segment with the longest segment (45.15 Mb) was SSC15, which contains 978 SNPs. In contrast, the shortest segment (1 Mb) only consisted of 265 SNPs (Figure 2A). In total, all ROH segments covered 13.4% of the entire genome.

The statistics of ROH number and length classified by ROH length are shown in Table 3. Among the three classes of ROH segments, the majority were short ROH segments (1–5 Mb), accounting for 78.46% of the total number of ROH segments, and it also had the highest genome coverage of 48.43% of the total length of all ROH segments. ROH_5–10 Mb_ had a lower quantity of 1,197 (15.94% of the total ROH number), and it made up 28.81% of the total ROH length. The number of long ROH segments was the lowest, only 5.57%, but it still had a coverage ratio of 22.76%.

**TABLE 3:** Descriptive statistics for ROH of different lengths (Mb): 1–5, 5–10, >10, and >1.

ROH length (Mb)	ROH number	Percent (%)	Mean length (Mb)	Standard deviation	Genome coverage (%)
1–5	5,893	78.49	2.32	1.05	6.49
5–10	1,197	15.94	6.79	1.29	3.86
>10	418	5.57	15.38	6.06	3.05
Total (>1)	7,508	100	3.76	3.71	13.40

For individuals, high variability was observed in both the number of ROH segments and the genome coverage; the former varied from 6 to 166, and the latter varied from 0.56 to 36.86%. For chromosomes, the number and coverage of ROH on each chromosome are shown in Figure 2B. These results suggest that SSC6 has the highest number of ROH segments of 688, and its coverage is 16.078%. In contrast, ROH segments in SSC12 have the lowest number of 215, and its coverage ratio is also the lowest (8.56%). SSC4 has the highest coverage ratio of 19.95%.

### Detection of Runs of Homozygosity Islands

**FIGURE 3:**
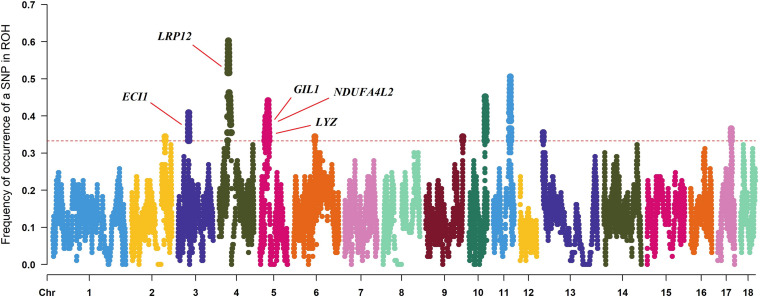
Frequency of occurrences of each SNP within ROH regions among all of the individuals. Horizontal red line represents the 33% threshold. Genes related to meat quality have been identified.

To identify the genomic regions with high ROH frequency, we first calculated the frequency of SNP occurrence in the ROH and selected the top 1% among those with the highest frequency (present in at least 33.3% of the samples) (Figure 3). There were four adjacent SNPs on SSC4 at ∼35 Mb with the highest frequency of 60.2%, which is similar to the findings of Wang et al. (2021), but no gene has been mapped in this region according to the reference genome (Sscrofa11.1). Neighboring selected SNPs that constitute regions with the highest frequency of ROH are called ROH islands. The genomic regions of the ROH islands detected in Laiwu pigs, distributed unevenly on different chromosomes with lengths varying from 0.031 to 14.4 Mb, are presented in Table 4. The longest ROH island was found to be on SSC6 and consisted of 320 contiguous SNPs, and the longest ROH cod-spot was on SSC4, containing 1,177 contagious SNPs.

**TABLE 4:** List of ROH islands observed in this Laiwu pig population.

Chromosome	Start (bp)	End (bp)	Length (bp)	Number of SNPs	Number of genes
2	125893724	126531130	637406	26	1
3	39414244	41549236	2134992	159	17
4	32235908	45876635	13640727	438	19
5	19810174	34227591	14417417	320	35
5	18987784	19153686	165902	8	0
5	18434915	18517600	82685	12	0
6	80696814	80728446	31632	7	0
9	138352276	139076532	724256	90	0
10	60710449	65079358	4368909	451	3
11	60646020	66330106	5684086	230	9
13	2559450	2749224	189774	19	4
17	51240101	51391328	151227	16	0
17	50326948	50888804	561856	42	1

### Candidate Genes Within Runs of Homozygosity Islands

**FIGURE 4:**
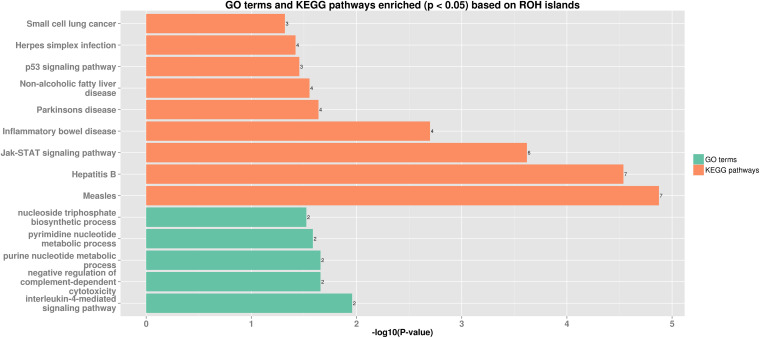
Bar plot of GO terms and KEGG pathways enriched (*p* < 0.05) based on ROH islands. Orange bars show enriched GO terms, and green bars show KEGG pathways.

Thirteen ROH islands were detected in our study, and there were 86 genes within those regions according to the University of California, Santa Cruz database (Supplementary Table 1). The position and number of the constituent SNPs and genes in those ROH islands are listed in Table 4. SSC5 has the most islands, and the longest island, which contains 320 SNPs and 35 genes, is also found between 19,810,174 and 34,227,591 bp. In some ROH islands that contain fewer contagious SNPs, no gene was detected. Candidate genes within all of the ROH islands detected were correlated with many economically important traits, such as meat quality (*ECI1*, *LRP12*, *NDUFA4L2*, *GIL1*, and *LYZ*), immunity capacity (*IL23A*, *STAT2*, *STAT6*, *TBK1*, *IFNG*, and *ITH2*), production (*DCSTAMP*, *RDH16*, and *GDF11*), and reproduction (*ODF1* and *CDK2*). All genes found were further subjected to GO and KEGG enrichment analyses. Six significant GO terms and nine significant KEGG pathways are shown in Figure 4 and Supplementary Table 2. Most terms and pathways were correlated with disease resistance and biosynthesis processes, and one KEGG pathway was related to lipid metabolism. In addition, we aligned all of these ROH islands to the pig quantitative trait loci (QTL) database and finally found eight QTLs related to the IMF trait.

## Discussion

### Characteristics of Runs of Homozygosity

In our study, the number of small ROH segments was the highest, whereas the number of medium and large segments was much lower, especially large segments (>10 Mb). Small ROH can reflect ancestry inbreeding history, whereas large segments are usually formed by recent inbreeding. These results indicated that both ancient and recent inbreeding events might affect this Laiwu population, but recent inbreeding was not frequent. However, it should be noted that the length of ROH may not always reflect the generations from inbreeding events that took place, as randomness exists during the formation of ROH, which can be affected by the dynamic rates of recombination, as well as the stochastic nature during gamete formation (Liu et al., 2010). Kirin et al. (2010) reported that short segments (<4 Mb) could also indicate the consequence of a decreased population size and bottleneck event.

The variability of the number of ROH among individuals was high, which coincides with the results of studies on cattle and sheep (Purfield et al., 2017; Peripolli et al., 2018). ROH number and coverage also differ from chromosome to chromosome, but the number of ROH tends to increase as the length of the chromosome increases. It has been reported that ROH are easy to form and inherit on some chromosomes but relatively difficult on other chromosomes, suggesting that some parts of the genome are less supportive of maintaining the accumulation of IBD than others (Nandolo et al., 2018). This phenomenon could be related to the selection. Chromosomes with high ROH coverage may suggest that they have been affected by positive selection, which will increase the accumulation of advantageous alleles and the neutral alleles genetically linked with them (Smith and Haigh, 1974). In contrast, the chromosome with low ROH coverage could indicate purifying selection, which decreases the number of homozygous deleterious alleles (Ferenčaković et al., 2013). In our study, some genomic regions of SSC4 and SSC12, which have the highest coverage and lowest coverage, respectively, may have been under natural or artificial selection.

The SNPs used to detect ROH regions were obtained from GGRS, a high-throughput reduced representation sequencing method in our study. It is a cost-effective approach and is designed for outbreed population, making it suitable for the population of Laiwu pigs in our research. However, the challenge is that the distribution of the data generated by it is unbalanced among individuals, which often causes a large portion of missing genotypes, which would impact subsequent analysis. To address this issue, imputation is inevitably used, and it has been included in the GGRS protocol. After imputation, we have also taken quality control measures, including filtering out the SNPs with a MAF < 0.05. This filter is necessary to ensure the precision of imputation (Wang et al., 2015).

There might be concerns about the necessity and appropriateness of imputation and the subsequent filtration with MAF, but several researchers have conducted studies that may help address such concerns. Imputed data have been used to detect ROH as found in several published works (Power et al., 2014; Chitneedi et al., 2017). It has been reported that using imputed data for ROH detection can produce comparable results with using the data that are restricted to genotyped SNPs only (Keller et al., 2012); Zhang et al. (2018) have also conducted the simulation to study the reliability of the use of imputed data in ROH detection and found that using imputed data with an average missing genotype rate of 0.7 could give a comparable amount of ROH detected compared with using data with no missing genotypes. Admittedly, removing monomorphic SNPs may reduce the chance to detect ROH, but it helps to exclude false-positive results. It has been reported that removing SNPs with low MAF (<0.05) before the analysis can minimize the trade-off between the exclusion of non-autozygous ROHs and the cost of missing shorter autozygous ROHs (Howrigan et al., 2011).

To date, few studies have been done comparing the performance of software used for ROH detection and the ways of determining appropriate parameters (Howrigan et al., 2011). Actually, a standard methodology for ROH detection is still ambiguous (Biscarini et al., 2020). In our study, we used PLINK software with default parameters to detect ROH; the same method has been applied in several published works (Peripolli et al., 2018; Xu et al., 2019).

### Runs of Homozygosity as a Tool of Inbreeding Estimation

The traditional method of estimating inbreeding coefficients is based on pedigree, and it is not as efficient as the method based on genomic information because errors usually exist in pedigree records. *F*_PED_ only indicates the expectation of homozygosis, whereas *F*_ROH_ and *F*_SNP_ can reflect the realized homozygous loci (Visscher et al., 2006). In our study, the value of *F*_PED_ was much lower than *F*_ROH_, which may be due to errors in the pedigree information. This may also be due to an inadequate pedigree, as *F*_ROH_ could suggest an inbreeding history 50 generations ago, but our pedigree only contains 8–10 generations of records. Some events, such as selection and mutation, were not involved in the method of estimating inbreeding coefficients, so the value of *F*_PED_ was likely to have a bias (Ceballos et al., 2018). In addition, our sequencing and genotyping method used reduced sequencing, which only covered 2–3% of the whole genome, so we may have overestimated the homozygosity of some regions and produced false-positive results during the process of identifying the ROH segments. Overestimating the number of ROH would also lead to an overestimation of inbreeding coefficients based on ROH.

In our study, the correlation between *F*_PED_ and *F*_ROH_ or *F*_PED_ and *F*_SNP_ was insignificant, which may be due to the inaccuracy of the pedigree. It has been reported that the correlation between *F*_PED_ and *F*_ROH_ would become stronger as the length of the ROH increase, as long ROH segments can reflect a recent inbreeding situation, which coincides with the trend found in our results (Magi et al., 2014). The correlation among the four indexes calculated by ROH was strong, especially between *F*_ROH_all_ and *F*_ROH1–5 Mb_ because the length of most ROH segments ranged from 1 to 5 Mb. The correlation between *F*_SNP_ and *F*_ROH_ was relatively strong, which may because the identifying of ROH was based on the results of SNP genotyping. *F*_SNP2_ correlates most strongly with *F*_ROH_ because they both directly reflect homozygosity.

### Analysis of Candidate Genes Within Runs of Homozygosity Islands

We identified and annotated several candidate genes contained by ROH islands and found they were related to various breeding traits, including meat quality, immunity, production, reproduction, etc. Because Laiwu pigs are characterized for high IMF contents, several candidate genes detected in our study were reported to be associated with lipid deposition and meat quality. *ECI1* expression was observed to have a positive correlation with IMF content (Lu et al., 2017). *LRP12* was reported to have highly significant associations with gluteus medius saturated fatty acid content in Duroc pigs (Melo et al., 2013). *NDUFA4L2* can affect metabolic enzyme activities, which has a direct impact on meat quality (Liu et al., 2016). *GIL1* negatively correlates with fat accumulation, and *LYZ* has been proven to be related to conductivity 24, pH1, and drip loss of porcine meat (Srikanchai et al., 2010). Some candidate genes relating to immunity were identified, such as *IL23A*, *STAT2*, *STAT6*, *TBK1*, and *IFNG*. It is noteworthy that *ITH2* was reported to be one of the putative PCV2 Cap-interacting host proteins by coimmunoprecipitation combined with liquid chromatography–mass spectrometry approach (Zhou et al., 2020), which may explain the strong ability of PCV2 resistance of Laiwu pigs. Some genes are related to production traits. *DCSTAMP* can affect feed gain ratio (Tao et al., 2017), and *RDH16* is also correlated with feed efficiency (Zhao et al., 2016). *GDF11* was proven to be the candidate gene involved in growth and body size in micro pigs (Son et al., 2020) and can raise the number of ribs in knock-out mice (Zhang et al., 2016). *ODF1*, participating in spermatogenesis and *CDK2*, affects the meiosis of oocytes, affecting the reproduction traits of pigs. *WIF1* could be a crucial mutation associated with ear size (Liang et al., 2019), which can correlate with the big ears of Laiwu pigs.

According to the Pig QTL database, several QTLs related to meat quality, immunity, the reproduction process, and other traits overlapping with the detected ROH islands are listed. In particular, some QTLs involved in the process of IMF deposition have already been reported: ID = 639 (Bink et al., 2000), ID = 145 (Gerbens et al., 2001), ID = 150, ID = 151, ID = 299 (Ovilo et al., 2002a,b), ID = 313 (de Koning et al., 1999), ID = 639 (Bink et al., 2000), and ID = 4169 (Ovilo et al., 2000).

## Conclusion

In summary, this study investigated the patterns of ROH and calculated inbreeding coefficients in 93 Laiwu pigs, which revealed how diversity evolved in the Laiwu population. We found that this population has been affected by historical inbreeding events, but recent inbreeding events are not frequent. Moreover, we identified 13 ROH islands putatively under natural and/or artificial selection harboring genes with molecular functions related to characteristic traits. We found that most genes contained by ROH islands were associated with disease resistance and biosynthesis processes. In addition, several genes related to economically important traits, such as meat quality, were also detected. These results may help us understand the characteristics of Laiwu pigs and provide insight for future breeding strategies.

## Data Availability Statement

The datasets presented in this study can be found in online repositories. The names of the repository/repositories and accession number(s) can be found in the article/Supplementary Material.

## Author Contributions

YP and QWe designed the experiment. YF, XH, ZX, and HS performed the experiment. QZ, PM, and ZZ developed some analysis software used in this study. YF wrote the manuscript with RC’s kind help. All authors have read and approved the final manuscript.

## Conflict of Interest

YS, ZQ, and QWe were employed by Breeding Company of Pigs, Laiwu, China. The remaining author declares that the research was conducted in the absence of any commercial or financial relationships that could be construed as a potential conflict of interest.

## Footnotes

1 http://genome.ucsc.edu/

2 https://david.ncifcrf.gov/

## References

[B1] Bink M. C. Te Pas M. F. Harders F. L. Janss L. L. (2000). A transmission/disequilibrium test approach to screen for quantitative trait loci in two selected lines of large white pigs. *Genet. Res.* 75 115–121. 10.1017/s0016672399004061 10740927

[B2] Biscarini F. Mastrangelo S. Catillo G. Senczuk G. Ciampolini R. (2020). Insights into genetic diversity, runs of homozygosity and heterozygosity-rich regions in maremmana semi-feral cattle using pedigree and genomic data. *Animals* 10:2285. 10.3390/ani10122285 33287320 PMC7761732

[B3] Bosse M. Megens H.-J. Madsen O. Paudel Y. Frantz L. A. F. Schook L. B. (2012). Regions of homozygosity in the porcine genome: consequence of demography and the recombination landscape. *PLoS Genet.* 8:e1003100. 10.1371/journal.pgen.1003100 23209444 PMC3510040

[B4] Browning B. L. Browning S. R. (2016). Genotype imputation with millions of reference samples. *Am. J. Hum. Genet.* 98 116–126. 10.1016/j.ajhg.2015.11.020 26748515 PMC4716681

[B5] Ceballos F. C. Joshi P. K. Clark D. W. Ramsay M. Wilson J. F. (2018). Runs of homozygosity: windows into population history and trait architecture. *Nat. Rev. Genet.* 19 220–234. 10.1038/nrg.2017.109 29335644

[B6] Chen Q. Ma Y. Yang Y. Chen Z. Liao R. Xie X. (2013). Genotyping by genome reducing and sequencing for outbred animals. *Plos One* 8:e67500. 10.1371/journal.pone.0067500 23874423 PMC3715491

[B7] Chitneedi P. Arranz J. Suarez-Vega A. García-Gámez E. Gutiérrez-Gil B. (2017). Estimations of linkage disequilibrium, effective population size and ROH-based inbreeding coefficients in Spanish Churra sheep using imputed high-density SNP genotypes. *Anim. Genet.* 48 436–446. 10.1111/age.12564 28543827

[B8] Coster A. Coster M. A. (2010). *Package ‘pedigree’. R package version.* 1.

[B9] Curik I. Ferenčaković M. Sölkner J. (2014). Inbreeding and runs of homozygosity: a possible solution to an old problem. *Livestock Sci.* 166 26–34. 10.1016/j.livsci.2014.05.034

[B10] de Koning D. J. Janss L. L. Rattink A. P. van Oers P. A. de Vries B. J. Groenen M. A. (1999). Detection of quantitative trait loci for backfat thickness and intramuscular fat content in pigs (*Sus scrofa*). *Genetics* 152 1679–1690. 10430592 10.1093/genetics/152.4.1679 PMC1460688

[B11] Falconer D. S. (1962). Falconer DS—introduction to quantitative genetics. *Population* 17 152–153. 10.2307/1525780

[B12] Ferencakovic M. Hamzic E. Gredler B. G. Curik I. Sölkner J. (2011). Runs of homozygosity reveal genome-wide autozygosity in the austrian fleckvieh cattle. *Agric. Conspectus Sci.* 76 325–329.

[B13] Ferenčaković M. Hamzić E. Gredler B. Solberg T. R. Klemetsdal G. Curik I. (2013). Estimates of autozygosity derived from runs of homozygosity: empirical evidence from selected cattle populations. *J. Anim. Breed Genet.* 130 286–293. 10.1111/jbg.12012 23855630

[B14] Frankham R. (1996). Introduction to quantitative genetics (4th edn). *Am. J. Hum. Genet.* 46:1231.

[B15] Gerbens F. Verburg F. J. Van Moerkerk H. T. Engel B. Buist W. Veerkamp J. H. (2001). Associations of heart and adipocyte fatty acid-binding protein gene expression with intramuscular fat content in pigs. *J. Anim. Sci.* 79 347–354. 10.2527/2001.792347x 11219443

[B16] Gibson J. Morton N. E. Collins A. (2006). Extended tracts of homozygosity in outbred human populations. *Hum. Mol. Genet.* 15 789–795. 10.1093/hmg/ddi493 16436455

[B17] Howard J. T. Pryce J. E. Baes C. Maltecca C. (2017). Invited review: Inbreeding in the genomics era: Inbreeding, inbreeding depression, and management of genomic variability. *J. Dairy Sci.* 100 6009–6024. 10.3168/jds.2017-12787 28601448

[B18] Howrigan D. P. Simonson M. A. Keller M. C. (2011). Detecting autozygosity through runs of homozygosity: a comparison of three autozygosity detection algorithms. *BMC Genom.* 12:460. 10.1186/1471-2164-12-460 21943305 PMC3188534

[B19] Keller M. C. Simonson M. A. Ripke S. Neale B. M. Gejman P. V. Howrigan D. P. (2012). Runs of homozygosity implicate autozygosity as a schizophrenia risk factor. *PLoS Genet.* 8:e1002656. 10.1371/journal.pgen.1002656 22511889 PMC3325203

[B20] Kirin M. McQuillan R. Franklin C. S. Campbell H. McKeigue P. M. Wilson J. F. (2010). Genomic runs of homozygosity record population history and consanguinity. *Plos One* 5:e13996. 10.1371/journal.pone.0013996 21085596 PMC2981575

[B21] Li H. Durbin R. (2010). Fast and accurate long-read alignment with burrows–wheeler transform. *Bioinformatics* 26 589–595. 10.1093/bioinformatics/btp698 20080505 PMC2828108

[B22] Li Y. P. Liu H. Wang P. F. Wang L. Y. Sun Y. Liu G. (2016). RNA-seq analysis reveals genes underlying different disease responses to porcine circovirus type 2 in pigs. *Plos One* 11:e0155502. 10.1371/journal.pone.0155502 27171165 PMC4865221

[B23] Liang J. Zhang Y. Wang L. Liu X. Yan H. Wang L. (2019). Molecular cloning of WIF1 and HMGA2 reveals ear-preferential expression while uncovering a missense mutation associated with porcine ear size in WIF1. *Anim. Genet.* 50 157–161. 10.1111/age.12759 30815903

[B24] Liu G. E. Hou Y. Zhu B. Cardone M. F. Jiang L. Cellamare A. (2010). Analysis of copy number variations among diverse cattle breeds. *Genom. Res.* 20 693–703. 10.1101/gr.105403.110 PMC2860171 20212021

[B25] Liu X. Trakooljul N. Murani E. Krischek C. Schellander K. Wicke M. (2016). Molecular changes in mitochondrial respiratory activity and metabolic enzyme activity in muscle of four pig breeds with distinct metabolic types. *J. Bioenerg. Biomemb.* 48 55–65. 10.1007/s10863-015-9639-3 26759028

[B26] Lu Y. F. Chen J. B. Zhang B. Li Q. G. Wang Z. X. Zhang H. (2017). Cloning, expression, and polymorphism of the ECI1 gene in various pig breeds. *J. Integr. Agric.* 16 1789–1799. 10.1016/s2095-3119(16)61624-6

[B27] MacLeod I. M. Larkin D. M. Lewin H. A. Hayes B. J. Goddard M. E. (2013). Inferring demography from runs of homozygosity in whole-genome sequence, with correction for sequence errors. *Mol. Biol. Evol.* 30 2209–2223. 10.1093/molbev/mst125 23842528 PMC3748359

[B28] Magi A. Tattini L. Palombo F. Benelli M. Gialluisi A. Giusti B. (2014). H3M2: detection of runs of homozygosity from whole-exome sequencing data. *Bioinformatics* 30 2852–2859. 10.1093/bioinformatics/btu401 24966365

[B29] McQuillan R. Leutenegger A. L. Abdel-Rahman R. Franklin C. S. Pericic M. Barac-Lauc L. (2008). Runs of homozygosity in european populations. *Am. J. Hum. Genet.* 83 359–372. 18760389 10.1016/j.ajhg.2008.08.007 PMC2556426

[B30] Melo C. Quintanilla R. Gallardo D. Zidi A. Jordana J. Diaz I. (2013). Association analysis with lipid traits of 2 candidate genes (LRP12 and TRIB1) mapping to a SSC4 QTL for serum triglyceride concentration in pigs. *J. Anim. Sci.* 91 1531–1537. 10.2527/jas.2012-5517 23408821

[B31] Nandolo W. Utsunomiya Y. T. Mészáros G. Wurzinger M. Khayadzadeh N. Torrecilha R. B. P. (2018). Misidentification of runs of homozygosity islands in cattle caused by interference with copy number variation or large intermarker distances. *Genet. Selec. Evol.* 50:43. 10.1186/s12711-018-0414-x PMC6106898 30134820

[B32] Nothnagel M. Lu T. T. Kayser M. Krawczak M. (2010). Genomic and geographic distribution of SNP-defined runs of homozygosity in europeans. *Hum. Mol. Genet.* 19 2927–2935. 10.1093/hmg/ddq198 20462934

[B33] Onzima R. B. Upadhyay M. R. Doekes H. P. Brito L. F. Bosse M. Kanis E. (2018). Genome-wide characterization of selection signatures and runs of homozygosity in ugandan goat breeds. *Front. Genet.* 9:318. 10.3389/fgene.2018.00318 30154830 PMC6102322

[B34] Ovilo C. Clop A. Noguera J. L. Oliver M. A. Barragán C. Rodriguez C. (2002a). Quantitative trait locus mapping for meat quality traits in an Iberian x Landrace F2 pig population. *J. Anim. Sci.* 80 2801–2808. 10.2527/2002.80112801x 12462246

[B35] Ovilo C. Oliver A. Noguera J. L. Clop A. Barragán C. Varona L. (2002b). Test for positional candidate genes for body composition on pig chromosome 6. *Genet. Sel. Evol.* 34 465–479. 12270105 10.1186/1297-9686-34-4-465 PMC2705456

[B36] Ovilo C. Pérez-Enciso M. Barragán C. Clop A. Rodríquez C. Oliver M. A. (2000). A QTL for intramuscular fat and backfat thickness is located on porcine chromosome 6. *Mamm Genome* 11 344–346. 10.1007/s003350010065 10754115

[B37] Patel R. K. Jain M. (2012). NGS QC toolkit: a toolkit for quality control of next generation sequencing data. *Plos One* 7:e30619. 10.1371/journal.pone.0030619 22312429 PMC3270013

[B38] Pemberton T. J. Absher D. Feldman Marcus W. Myers Richard M. Rosenberg Noah A. Li Jun Z. (2012b). Genomic patterns of homozygosity in worldwide human populations. *Am. J. Hum. Genet.* 91 275–292. 10.1016/j.ajhg.2012.06.014 22883143 PMC3415543

[B39] Pemberton T. J. Absher D. Feldman M. W. Myers R. M. Rosenberg N. A. Li J. Z. (2012a). Genomic patterns of homozygosity in worldwide human populations. *Am. J. Hum. Genet.* 91 275–292. 10.1016/j.ajhg.2012.06.014 22883143 PMC3415543

[B40] Peripolli E. Munari D. P. Silva M. V. G. B. Lima A. L. F. Irgang R. Baldi F. (2017). Runs of homozygosity: current knowledge and applications in livestock. *Anim. Genet.* 48 255–271. 10.1111/age.12526 27910110

[B41] Peripolli E. Stafuzza N. B. Munari D. P. Lima A. L. F. Irgang R. Machado M. A. (2018). Assessment of runs of homozygosity islands and estimates of genomic inbreeding in Gyr (Bos indicus) dairy cattle. *BMC Genom.* 19:34. 10.1186/s12864-017-4365-3 29316879 PMC5759835

[B42] Power R. A. Keller M. C. Ripke S. Abdellaoui A. Wray N. R. Sullivan P. F. (2014). A recessive genetic model and runs of homozygosity in major depressive disorder. *Am. J. Med. Genet. B Neuropsychiatr. Genet.* 165 157–166. 10.1002/ajmg.b.32217 24482242 PMC4234115

[B43] Purcell S. Neale B. Todd-Brown K. Thomas L. Ferreira M. A. R. Bender D. (2007). PLINK: a tool set for whole-genome association and population-based linkage analyses. *Am. J. Hum. Genet.* 81 559–575. 10.1086/519795 17701901 PMC1950838

[B44] Purfield D. C. Berry D. P. McParland S. Bradley D. G. (2012). Runs of homozygosity and population history in cattle. *BMC Genet.* 13:70. 10.1186/1471-2156-13-70 22888858 PMC3502433

[B45] Purfield D. C. McParland S. Wall E. Berry D. P. (2017). The distribution of runs of homozygosity and selection signatures in six commercial meat sheep breeds. *Plos One* 12:e0176780. 10.1371/journal.pone.0176780 28463982 PMC5413029

[B46] Ron M. Blanc Y. Band M. Ezra E. Weller J. I. (1996). Misidentification rate in the israeli dairy cattle population and its implications for genetic improvement. *J. Dairy Sci.* 79 676–681. 10.3168/jds.s0022-0302(96)76413-5 8744233

[B47] Smith J. M. Haigh J. (1974). The hitch-hiking effect of a favourable gene. *Genet. Res.* 23 23–35. 10.1017/s0016672300014634 4407212

[B48] Son D. Hwang N. H. Chung W. H. Seong H. Lim H. Cho E. S. (2020). Whole-genome resequencing analysis of 20 Micro-pigs. *Genes Genom.* 42 263–272. 10.1007/s13258-019-00891-x 31833050

[B49] Srikanchai T. Murani E. Wimmers K. Ponsuksili S. (2010). Four loci differentially expressed in muscle tissue depending on water-holding capacity are associated with meat quality in commercial pig herds. *Mol. Biol. Rep.* 37 595–601. 10.1007/s11033-009-9856-0 19823956

[B50] Szmatoła T. Gurgul A. Jasielczuk I. Ząbek T. Ropka-Molik K. Litwińczuk Z. (2019). A comprehensive analysis of runs of homozygosity of eleven cattle breeds representing different production types. *Animals (Basel)* 9:1024. 10.3390/ani9121024 31775271 PMC6941163

[B51] Tao X. Liang Y. Yang X. M. Pang J. H. Zhong Z. J. Chen X. H. (2017). Transcriptomic profiling in muscle and adipose tissue identifies genes related to growth and lipid deposition. *Plos One* 12:e0184120. 10.1371/journal.pone.0184120 28877211 PMC5587268

[B52] VanRaden P. M. (2008). Efficient methods to compute genomic predictions. *J. Dairy Sci.* 91 4414–4423. 10.3168/jds.2007-0980 18946147

[B53] Visscher P. M. Medland S. E. Ferreira M. A. R. Morley K. I. Zhu G. Cornes B. K. (2006). Assumption-free estimation of heritability from genome-wide identity-by-descent sharing between full siblings. *PLoS Genet.* 2:e41. 10.1371/journal.pgen.0020041 16565746 PMC1413498

[B54] Wang X. Zhang H. Huang M. Tang J. Yang L. Yu Z. (2021). Whole-genome SNP markers reveal conservation status, signatures of selection, and introgression in chinese laiwu pigs. *Evol. Appl.* 14 383–398. 10.1111/eva.13124 33664783 PMC7896721

[B55] Wang Z. Chen Q. Yang Y. Liao R. Zhao J. Zhang Z. (2015). Genetic diversity and population structure of six chinese indigenous pig breeds in the taihu Lake region revealed by sequencing data. *Anim. Genet.* 46 697–701. 10.1111/age.12349 26373882 PMC5049631

[B56] Wright S. (1922). Coefficients of inbreeding and relationship. *Am. Naturalist* 56 330–338. 10.1086/279872

[B57] Wright S. (1948). Genetics of populations. *Encyclopaedia Britannica* 10 111–112.

[B58] Xiao Q. Zhang Z. Sun H. Wang Q. Pan Y. (2017). Pudong white pig: a unique genetic resource disclosed by sequencing data. *Animal* 11 1117–1124. 10.1017/s1751731116002494 27903314

[B59] Xu Z. Sun H. Zhang Z. Zhao Q. Olasege B. S. Li Q. (2019). Assessment of autozygosity derived from runs of homozygosity in jinhua pigs disclosed by sequencing data. *Front. Genet.* 10:274. 10.3389/fgene.2019.00274 30984245 PMC6448551

[B60] Yang H. Huang X. Zeng Z. Zhang W. Liu C. Fang S. (2015). Genome-wide association analysis for blood lipid traits measured in three pig populations reveals a substantial level of genetic heterogeneity. *Plos One* 10:e0131667. 10.1371/journal.pone.0131667 26121138 PMC4488070

[B61] Yang J. Lee S. H. Goddard M. E. Visscher P. M. (2011). GCTA: a tool for genome-wide complex trait analysis. *Am. J. Hum. Genet.* 88 76–82. 10.1016/j.ajhg.2010.11.011 21167468 PMC3014363

[B62] Zanella R. Peixoto J. O. Cardoso F. F. Cardoso L. L. Biegelmeyer P. Cantão M. E. (2016). Genetic diversity analysis of two commercial breeds of pigs using genomic and pedigree data. *Genet. Selec. Evol.* 48:24. 10.1186/s12711-016-0203-3 PMC4812646 27029213

[B63] Zhan H. Zhang S. Zhang K. Peng X. Xie S. Li X. (2020). Genome-wide patterns of homozygosity and relevant characterizations on the population structure in piétrain pigs. *Genes (Basel)* 11:577. 10.3390/genes11050577 32455573 PMC7291003

[B64] Zhang L. C. Yue J. W. Pu L. Wang L. G. Liu X. Liang J. (2016). Genome-wide study refines the quantitative trait locus for number of ribs in a large white x minzhu intercross pig population and reveals a new candidate gene. *Mol. Genet. Genom.* 291 1885–1890. 10.1007/s00438-016-1220-1 27307002

[B65] Zhang Q. Guldbrandtsen B. Bosse M. Lund M. S. Sahana G. (2015). Runs of homozygosity and distribution of functional variants in the cattle genome. *BMC Genom.* 16:542. 10.1186/s12864-015-1715-x 26198692 PMC4508970

[B66] Zhang Z. Zhang Q. Xiao Q. Sun H. Gao H. Yang Y. (2018). Distribution of runs of homozygosity in chinese and western pig breeds evaluated by reduced-representation sequencing data. *Anim. Genet.* 49 579–591. 10.1111/age.12730 30324759

[B67] Zhao Y. X. Hou Y. Liu F. Liu A. Jing L. Zhao C. Z. (2016). Transcriptome analysis reveals that vitamin A metabolism in the liver affects feed efficiency in pigs. *G3-Genes Genom. Genet.* 6 3615–3624. 10.1534/g3.116.032839 27633790 PMC5100860

[B68] Zhou J. W. Li H. Y. Yu T. Q. Li J. R. Dong W. R. Ojha N. K. (2020). Protein interactions network of porcine circovirus type 2 capsid with host proteins. *Front. Microbiol.* 11:1129. 10.3389/fmicb.2020.01129 32582087 PMC7283462

