# Supplementary material for: Genome-Wide Detection of Runs of Homozygosity in Laiwu Pigs Revealed by Sequencing Data
Source: Front Genet. 2021 Apr 29;12:629966. doi: 10.3389/fgene.2021.629966 (PMC8116706; doi:10.3389/fgene.2021.629966)
Supplement: Supplementary file 1 [file Data_Sheet_1.doc]

Supplementary Material

**Genome-wide detection of runs of homozygosity in Laiwu pigs revealed by sequencing data**

Yifei Fang^1^, Xinyu Hao^1^, Zhong Xu^1^, Hao Sun^1^, Qingbo Zhao^1^, Qamar Raza Qadri^1^, Rui Cao^1^, Zhe Zhang^2^, Peipei Ma^1^, Qishan Wang^2*^, Yuchun Pan^2*^

^1^*Department of Animal Science, School of Agriculture and Biology, Shanghai Jiao Tong University, Shanghai 200240, PR China*

*^2^Department of Animal Breeding and Reproduction, College of Animal Sciences, Zhejiang University, Hangzhou 310058, PR China*

***Correspondence:**

Yuchun Pan;

Qishan Wang

E-mail: [panyuchun1963@aliyun.com](mailto:panyuchun1963@aliyun.com)

[wangqishan@zju.edu.cn](mailto:wangqishan@sjtu.edu.cn)

**Table S1.** List of 87 potential candidate genes within ROH islands in the Laiwu pigs.

| Chr | Gene Start (bp) | Gene End (bp) | Gene Name | Ensembl Gene ID |
| --- | --- | --- | --- | --- |
| 2 | 125889164 | 125956864 | *SNCAIP* | ENSSSCG00035059291 |
| 3 | 39750741 | 39758516 | *ECI1* | ENSSSCG00015080865 |
| 3 | 39779394 | 39779490 | *MIR1842* | ENSSSCG00000042435 |
| 3 | 39849608 | 39899455 | *PKD1* | ENSSSCG00000008041 |
| 3 | 39942370 | 39952824 | *SLC9A3R2* | ENSSSCG00000008038 |
| 3 | 39957041 | 39957792 | *NPW* | ENSSSCG00000048627 |
| 3 | 40017160 | 40021736 | *MSRB1* | ENSSSCG00000032932 |
| 3 | 40174798 | 40177789 | *IGFALS* | ENSSSCG00000008013 |
| 3 | 40178416 | 40184585 | *NUBP2* | ENSSSCG00000008014 |
| 3 | 40194077 | 40195316 | *MRPS34* | ENSSSCG00000008016 |
| 3 | 40195639 | 40196872 | *NME3* | ENSSSCG00000008017 |
| 3 | 40245472 | 40256960 | *JPT2* | ENSSSCG00000008019 |
| 3 | 40556555 | 40568941 | *UBE2I* | ENSSSCG00000021560 |
| 3 | 40596407 | 40598426 | *LOC396700* | ENSSSCG00000008032 |
| 3 | 40616642 | 40618155 | *MCT7* | ENSSSCG00000021222 |
| 3 | 40756464 | 40758111 | *SSTR5* | ENSSSCG00000033234 |
| 3 | 41100875 | 41106370 | *RHOT2* | ENSSSCG00045016397 |
| 3 | 41306172 | 41310312 | *NME4* | ENSSSCG00000007986 |
| 4 | 32701945 | 32779159 | *LRP12* | ENSSSCG00000006039 |
| 4 | 32913154 | 32928235 | *DCSTAMP* | ENSSSCG00000006043 |
| 4 | 33811158 | 33844556 | *FZD6* | ENSSSCG00000006052 |
| 4 | 33894239 | 33983920 | *BAALC* | ENSSSCG00000006053 |
| 4 | 34033942 | 34065197 | *ATP6V1C1* | ENSSSCG00000006054 |
| 4 | 34226750 | 34257261 | *AZIN1* | ENSSSCG00000023873 |
| 4 | 34430131 | 34435989 | *KLF10* | ENSSSCG00000027480 |
| 4 | 34511076 | 34520601 | *ODF1* | ENSSSCG00000006056 |
| 4 | 35273635 | 35369393 | *NCALD* | ENSSSCG00000006059 |
| 4 | 35979826 | 36007835 | *YWHAZ* | ENSSSCG00000006062 |
| 4 | 36400637 | 36443798 | *ANKRD46* | ENSSSCG00000006065 |
| 4 | 36690082 | 36724243 | *RNF19A* | ENSSSCG00000006066 |
| 4 | 37074618 | 37089364 | *COX6C* | ENSSSCG00005036737 |
| 4 | 38716879 | 38720415 | *RPL30* | ENSSSCG00000006081 |
| 4 | 39055815 | 39113305 | *MTDH* | ENSSSCG00000006084 |
| 4 | 39492662 | 39967556 | *CPQ* | ENSSSCG00000006087 |
| 4 | 40003617 | 40129483 | *SDC2* | ENSSSCG00000006088 |
| 4 | 40403303 | 40409079 | *UQCRB* | ENSSSCG00060073316 |
| 4 | 41804883 | 41821839 | *CCNE2* | ENSSSCG00000006095 |
| 5 | 21191387 | 21199024 | *GDF11* | ENSSSCG00000000363 |
| 5 | 21264437 | 21273235 | *DNAJC14* | ENSSSCG00000000366 |
| 5 | 21331649 | 21355531 | *DGKA* | ENSSSCG00000000370 |
| 5 | 21365829 | 21372003 | *CDK2* | ENSSSCG00000037597 |
| 5 | 21395298 | 21399716 | *SUOX* | ENSSSCG00000000376 |
| 5 | 21431655 | 21433691 | *RPS26* | ENSSSCG00045036682 |
| 5 | 21559324 | 21562418 | *MYL6* | ENSSSCG00000039506 |
| 5 | 21671200 | 21700563 | *CS* | ENSSSCG00000035686 |
| 5 | 21707833 | 21711292 | *CNPY2* | ENSSSCG00000000394 |
| 5 | 21732415 | 21733981 | *IL23A* | ENSSSCG00000033520 |
| 5 | 21733029 | 21748960 | *STAT2* | ENSSSCG00000000396 |
| 5 | 21759669 | 21760753 | *APOF* | ENSSSCG00000000398 |
| 5 | 22064250 | 22089441 | *PRIM1* | ENSSSCG00000026055 |
| 5 | 22251750 | 22257746 | *RDH16* | ENSSSCG00000000419 |
| 5 | 22324929 | 22332815 | *TAC3* | ENSSSCG00000000418 |
| 5 | 22406363 | 22421448 | *STAT6* | ENSSSCG00000040435 |
| 5 | 22540481 | 22542661 | *NDUFA4L2* | ENSSSCG00000027621 |
| 5 | 22736707 | 22747420 | *GLI1* | ENSSSCG00000000443 |
| 5 | 22785581 | 22787017 | *DDIT3* | ENSSSCG00000044553 |
| 5 | 23039205 | 23041959 | *CDK4* | ENSSSCG00000025092 |
| 5 | 23053890 | 23058470 | *CYP27B1* | ENSSSCG00000028637 |
| 5 | 23111365 | 23132811 | *CTDSP2* | ENSSSCG00000031707 |
| 5 | 23112743 | 23112827 | *MIR26A* | ENSSSCG00000022286 |
| 5 | 27252801 | 27252886 | *MIRLET7I* | ENSSSCG00000019827 |
| 5 | 28883289 | 28929264 | *TBK1* | ENSSSCG00000031103 |
| 5 | 29450441 | 29543913 | *WIF1* | ENSSSCG00000030998 |
| 5 | 29596333 | 29672700 | *LEMD3* | ENSSSCG00000000469 |
| 5 | 31452346 | 31452427 | *MIR9808* | ENSSSCG00000038564 |
| 5 | 32477905 | 32482670 | *IFNG* | ENSSSCG00000032963 |
| 5 | 33105717 | 33135592 | *MDM2* | ENSSSCG00000000488 |
| 5 | 33525757 | 33555558 | *CPSF6* | ENSSSCG00000038031 |
| 5 | 33612592 | 33618953 | *LYZ* | ENSSSCG00015067276 |
| 5 | 33725455 | 33849957 | *FRS2* | ENSSSCG00000000493 |
| 5 | 33858536 | 33873596 | *CCT2* | ENSSSCG00000000496 |
| 10 | 63369257 | 63387963 | *GATA3* | ENSSSCG00000011125 |
| 10 | 63635205 | 63672200 | *ITIH2* | ENSSSCG00000011128 |
| 10 | 64948905 | 64994529 | *IL2RA* | ENSSSCG00000022849 |
| 11 | 60734655 | 60734732 | *MIR17* | ENSSSCG00000018617 |
| 11 | 60734790 | 60734882 | *MIR18A* | ENSSSCG00000019535 |
| 11 | 60734938 | 60735020 | *MIR19A* | ENSSSCG00000019897 |
| 11 | 60735108 | 60735179 | *MIR20A* | ENSSSCG00000019883 |
| 11 | 60735237 | 60735317 | *MIR19B-1* | ENSSSCG00000019902 |
| 11 | 60735357 | 60735437 | *MIR92A-1* | ENSSSCG00000018681 |
| 11 | 63587529 | 63635454 | *DCT* | ENSSSCG00000009490 |
| 11 | 65121626 | 65145002 | *CLDN10* | ENSSSCG00000009500 |
| 11 | 65230144 | 65299084 | *DNAJC3* | ENSSSCG00000026082 |
| 13 | 2638972 | 2654829 | *METTL6* | ENSSSCG00000011190 |
| 13 | 2654828 | 2671140 | *EAF1* | ENSSSCG00000026622 |
| 17 | 50744955 | 50796466 | *CSE1L* | ENSSSCG00000025534 |

**Table S2.** GO terms and KEGG pathways enriched (p < 0.05) based on ROH islands.

| ID | Term | P value | Count |
| --- | --- | --- | --- |
| GO:0000082 | G1/S transition of mitotic cell cycle | 0.0061 | 3 |
| GO:0035771 | interleukin-4-mediated signaling pathway | 0.011 | 2 |
| GO:1903660 | negative regulation of complement-dependent cytotoxicity | 0.022 | 2 |
| GO:0006163 | purine nucleotide metabolic process | 0.022 | 2 |
| GO:0006220 | pyrimidine nucleotide metabolic process | 0.026 | 2 |
| GO:0009142 | nucleoside triphosphate biosynthetic process | 0.03 | 2 |
| ssc05162 | Measles | 1.33355E-05 | 7 |
| ssc05161 | Hepatitis B | 0.000029 | 7 |
| ssc04630 | Jak-STAT signaling pathway | 0.00024015 | 6 |
| ssc05321 | Inflammatory bowel disease | 0.002 | 4 |
| ssc05012 | Parkinson's disease | 0.023 | 4 |
| ssc04932 | Non-alcoholic fatty liver disease | 0.028 | 4 |
| ssc04115 | p53 signaling pathway | 0.035 | 3 |
| ssc05168 | Herpes simplex infection | 0.038 | 4 |
| ssc05222 | Small cell lung cancer | 0.048 | 3 |
